# The Association between Female Reproductive Factors and Open-Angle Glaucoma in Korean Women: The Korean National Health and Nutrition Examination Survey V

**DOI:** 10.1155/2018/2750786

**Published:** 2018-06-20

**Authors:** Yong Un Shin, Eun Hee Hong, Min Ho Kang, Heeyoon Cho, Mincheol Seong

**Affiliations:** Department of Ophthalmology, Hanyang University College of Medicine, Seoul, Republic of Korea

## Abstract

**Purpose:**

We investigated associations between female reproductive factors and open-angle glaucoma (OAG) in Korean females using the Korea National Health and Nutrition Examination Survey (KNHANES).

**Methods:**

A nationwide, population-based, cross-sectional study was conducted. We enrolled 23,376 participants from the KNHANES who had undergone ophthalmologic exams from 2010 through 2012. Associations between undiagnosed OAG and female reproductive factors such as age at menarche and menopause, parity, history of lactation, and administration of oral contraceptives (OC) or hormone replacement therapy (HRT) were determined using stepwise logistic regression analyses.

**Results:**

Of the enrolled participants, 6,860 participants (397 with OAG and 6,463 without OAG) met our study criteria and were included in the analyses. In the multivariate logistic regression analysis after adjusting for all potential confounding factors, only early menopause (younger than 45 years) was significantly associated with OAG in participants with natural menopause (OR 2.28, 95% CI 1.17–4.46). Age at menarche, parity, history of lactation, and administration of OC or HRT were not significantly associated with OAG.

**Conclusions:**

Only early menopause was associated with an increased risk of OAG in our study, in contrast to previous Western studies reporting both early menopause and late menarche as associated factors.

## 1. Introduction

Glaucoma, the second leading cause of blindness worldwide [1], is a neurodegenerative disease characterized by the loss of retinal ganglion cells (RGCs) and their axons [2]. Among known risk factors, including elevated intraocular pressure (IOP), family history, older age and African American ethnicity [1], elevated IOP is the major risk factor for developing the disease and the only confirmed modifiable risk factor. However, visual field loss and RGC death also occur and progress in cases of controlled or normal IOP [3]. Furthermore, therapies aimed at lowering IOP are not always successful and early detection does not always lead to prevention of visual impairment because of the lack of highly effective treatments. Accordingly, complementary strategies targeting alternate modifiable glaucoma risk factors other than IOP are needed to manage and screen for the disease.

There is some emerging evidence that estrogen plays an important role in the pathogenesis of open-angle glaucoma (OAG). Several previous population-based studies conducted in Caucasians have found a relationship between factors associated with exposure to estrogen such as age at menopause or menarche, use of hormone replacement therapy (HRT) in postmenopausal women, or use of oral contraceptives (OC) and the risk of OAG [4–7]. However, only two population-based studies have examined associations between female reproductive factors and OAG in Asian populations: one in a rural Indian population [8] and the other in a population with Malay ethnicity [9].

The Korea National Health and Nutrition Examination Survey (KNHANES) includes extensive data on health and socioeconomic status from a large population in Korea [10]. Using the raw data from KNHANES, we conducted a population-based study to describe the effects of female reproductive factors on OAG in Korean females.

## 2. Materials and Methods

### 2.1. Study Population

The KNHANES is a population-based, cross-sectional survey conducted in South Korea by the Division of Chronic Disease Surveillance of the Korea Centers for Disease Control and Prevention and the Korean Ministry of Health and Welfare. The KNHANES uses a complex, multistage, stratified, probability-clustered sampling method to analyze a representative, civilian, noninstitutionalized South Korean population. The details of the KNHANES have been described previously [11, 12]. We examined data obtained from a representative sample in the period between 2010 and 2012, using the fifth Korea National Health and Nutrition Examination Survey (KNHANES-V, 2010–2012), which employed stratified multistage sampling based on geographical area, sex, and age group [11].

**Figure 1:**
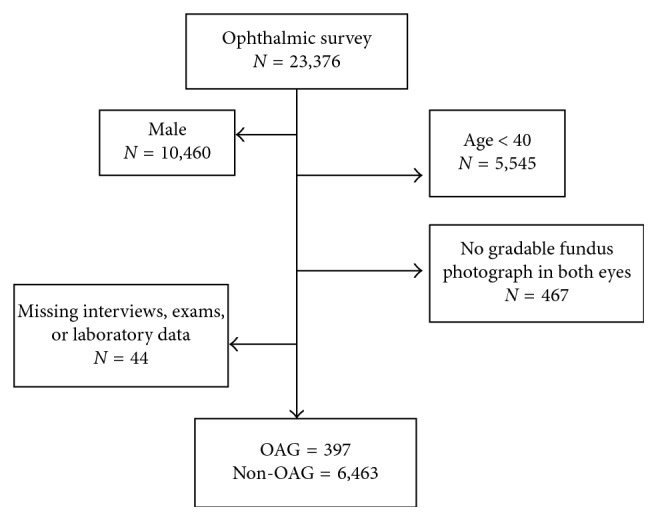
Flow chart for selection of the study participants. A total of 16,516 participants were excluded due to male sex, age < 40 years old, and ungradable.

The KNHANES consists of three parts: the Health Interview Survey, the Health Examination Survey, and the Nutritional Survey. Ophthalmologic interviews and exams were only conducted in participants ≥19 years old. The present study included the data from a randomly chosen eye of 23,376 individuals from the KNHANES between 2010 and 2012 who met the following inclusion criteria: were women 40 years of age or older; received the ophthalmology survey; and had a gradable fundus photograph and frequency-doubling technology (FDT) perimetry test result for at least one eye. Participants with missing data or unreliable examination results were excluded (Figure 1).

The survey adhered to the tenets of the Declaration of Helsinki, and written informed consent was obtained from all KNHANES participants. The survey protocol and study design were reviewed and approved by the Institutional Review Board (IRB) of the Korean Centers for Diseases Control and Prevention.

### 2.2. Measurements

We obtained data from the Health Interview Survey and Health Examination Survey. In the Health Interview Survey, participants responded to questions about female health: age at menarche, age at menopause and whether it was natural or artificial, parity, history of lactation, administration and duration of OC, and administration of HRT.

The Health Examination Survey included blood pressure (BP), physical examination, and basic laboratory tests. Physical examinations were performed by trained investigators following a standardized procedure. Body weight and height were measured in light indoor clothing without shoes to the nearest 0.1 kg and 0.1 cm. Body mass index (BMI) was calculated as the ratio of weight/height [2] (kg/m^2^). Systolic blood pressure (SBP) and diastolic blood pressure (DBP) were measured on the right arm using a standard Mercury sphygmomanometer (Baumanometer, Baum, Copiague, NY, USA). We calculated final blood pressure by averaging the second and third blood pressure measurements. For the routine blood test, blood samples were collected after at least an eight-hour fasting period and analyzed within 24 hours after transport to a certified laboratory using a Hitachi 7600-110 chemistry analyzer (Hitachi, Tokyo, Japan). Fasting plasma glucose (FPG) was also analyzed.

Ophthalmology-focused interviews were performed using self-reported questionnaires, including past or current medical or surgical conditions relevant to ophthalmology, such as a history of cataract surgery. Subjects underwent a detailed eye examination, which included visual acuity measurements; autorefraction using an autorefractor keratometer (KR8800, Topcon, Tokyo, Japan); slit-lamp examination, including assessment of peripheral anterior chamber depth by the Van Herick method (Haag-Streit model BQ-900; Haag-Streit AG, Koeniz, Switzerland); IOP, measured by Goldmann applanation tonometry (GAT; Haag-Streit, Haag-Streit AG) by ophthalmologists; and fundus photographs with a nonmydriatic 45° digital fundus camera (TRC-NW6S, Topcon) and a Nikon D-80 digital camera (Nikon, Tokyo, Japan). These ophthalmologic examinations were performed in a mobile examination unit by a trained ophthalmologist or ophthalmology resident. In addition, visual field testing using the N-30-1 screening program (Humphrey Matrix frequency-doubling perimeter, Carl Zeiss Meditec, Inc., Dublin, CA, USA) was performed on participants who had elevated IOP (≥22 mmHg), a horizontal or vertical cup-to-disc ratio ≥ 0.5, violation of the ISNT rule (neuroretinal rim broadest in the inferior area in the normal eye, followed by the superior, nasal, and temporal areas), an optic disc hemorrhage, or a retinal nerve fiber layer (RNFL) defect. The test location was deemed abnormal if not identified on two attempts at a contrast level identified by 99% of the healthy population. Frequency doubling technology was repeated if the rate of fixation errors was more than 0.33 or if the false-positive rate was greater than 0.33. An FDT with greater than a 0.33 rate of fixation errors or 0.33 rate of false positives was deemed invalid and was not used as criteria for glaucoma classification and required either International Society of Geographical and Epidemiological Ophthalmology (ISGEO) category 2 criteria (described below) for a glaucoma diagnosis.

### 2.3. Variable Definition

We defined several new variables in this study from KNHANES raw data. Early menarche was defined as age at menarche younger than or at 12 years old and late menarche as older than or at 17 years old. Early menopause was defined as age at menopause younger than 45 years old. The reproductive years were defined as the period between age at menarche and age at menopause. The duration of OC use was categorized into 2 groups: <3 years of OC use and ≥3 years of OC use, as in a previous study [13]. Hypertension presence was defined as systolic pressure ≥ 140 mmHg, diastolic pressure ≥ 90 mmHg, or a current prescription for antihypertensive medication. Diabetes presence was defined as fasting glucose ≥126 mg/dL or a current prescription for antiglycemic medication. Myopia was defined as a spherical equivalent less than or equal to −1.00 diopters (D).

The definition of open-angle glaucoma (OAG) was based on ISGEO criteria and a previous study [14, 15]. Patients were defined as having open-angle glaucoma if an open angle (peripheral anterior chamber depth > 1/4 corneal thickness) was present along with any one of the following category diagnostic criteria. Category I criteria: the presence of FDT testing results and fixation error and false-positive error ≤ 1: (i) loss of neuroretinal rim with vertical or horizontal cup : disc ratio ≥ 0.6, or asymmetry of vertical cup : disc ratio ≥ 0.2 (both values determined by ≥97.5th percentile for the normal population in the KNHANES), or a retinal nerve fiber layer defect; and (ii) the presence of an abnormal FDT testing result (at least one location of reduced sensitivity). Category II criteria: the absence of FDT testing results or fixation error or false-positive error ≥ 2: (i) loss of neuroretinal rim with vertical cup: disc ratio ≥ 0.9 or asymmetry of vertical cup: disc ratio ≥ 0.3 (both values determined by ≥99.5th percentile for the normal population in the KNHANES), or (ii) the presence of a retinal nerve fiber layer defect with violation of the ISNT rule (the neuroretinal rim thickness order of inferior > superior > nasal > temporal). Category III (when no visual field testing or optic disc exam was available) required a visual acuity < 20/400 and an IOP greater than 21 mmHg.

### 2.4. Statistical Analysis

Statistical analyses for a complex sampling design were performed using SPSS 21.0 Version (IBM, Armonk, NY, USA). According to statistical guidelines from the Korea Centers for Disease Control and Prevention, survey sample weights were used in all analyses to produce an integrated new dataset from the 3-year data that was representative of the noninstitutionalized civilian Korean population. Baseline characteristics of the study participants were expressed as either weighted means ±standard error (SE) for continuous variables or numbers and percentage (%) ± SE for categorical variables as appropriate for total participants and were compared using the Student's *t*-test and the chi-square test, respectively, between the OAG and non-OAG groups. To determine which factors had significant associations with OAG, potential associated factors for OAG were investigated via univariate logistic regression analysis. This step was performed in both the study population as a whole and participants with natural menopause, respectively, and in each group we also performed age-adjusted analyses. In the next step, multivariate adjusted logistic regression analyses were conducted to examine the odds ratio (OR) and 95% confidence interval (CI) for the association between OAG and associated factors, adjusting for age and all other confounders (DM, HTN, myopia, BMI, OC, and HRT in Model I; addition of early menopause, age at menarche, lactation, and parity in Model II). Factors that yielded a *P* value < 0.05 were considered statistically significant.

## 3. Results

### 3.1. Baseline Characteristics

After application of exclusion criteria, 6,860 participants were eligible for this study (397 participants with OAG and 6,463 participants without OAG). Of all the participants who underwent an ophthalmic survey, we excluded 16,516 participants due to male sex, age < 40 years old, ungradable fundus image on either eye or missing survey data (Figure 1). Overall, the mean age of all participants in this study was 56.09 ± 0.2 years. Among the included participants, 36.6 ± 0.8% had hypertension and 11.0 ± 0.5% had diabetes. The mean body mass index was 24.0 ± 0.06 kg/m^2^. Among female reproductive factors, mean age at menarche and menopause was 15.5 ± 0.1 years and 48.7 ± 0.1 years, respectively. The mean number of parity and reproductive years was 4.3 ± 0.01 and 32.7 ± 0.1 years, respectively. Natural menopause occurred in 86.2 ± 0.7% of participants with menopause. The percentage of participants who took OC and HRT was 17.6 ± 0.6% and 10.9 ± 0.5%, respectively. A history of lactation was reported by 82.6% of participants. The percentage of participants with myopia was 23.0 ± 0.7%. Differences in demographic characteristics according to the presence of OAG are shown in Table 1. Participants with OAG were more likely to be older (62.7 ± 0.8 versus 55.8 ± 0.2 years, *P* < 0.001) and to have hypertension (53.8 ± 3.4 versus 35.7 ± 0.8%, *P* < 0.001) or diabetes (20.4 ±2.8 versus 10.5 ± 0.5%, *P* < 0.001) than participants without OAG. Among female reproductive factors, both the proportion of participants with early menopause and parity were significantly different between participants with and without OAG. There was no significant difference in the proportion of age at menarche, natural menopause, OC use, HRT, or history of lactation. The proportion of participants with myopia was similar between the two groups.

### 3.2. Association of OAG with Female Reproductive Factors

The prevalences of OAG (presented as mean ± standard error) for the different age groups, 40 to 49 years, 50 to 59 years, 60 to 69 years, and 70 years or older, were 2.4 ± 0.4%, 3.8 ± 0.5%, 6.4 ± 0.7%, and 9.9 ± 0.9%, respectively.  Table 2 shows OAG-associated factors as determined by logistic regression analysis. In univariate logistic regression analyses, the following factors were significantly associated with OAG: old age (OR 1.04 per 1 year, 95% CI 1.03–1.06), early menopause (OR 1.56, 95% CI 1.03–2.35), parity (OR 1.15 per one pregnancy, 95% CI 1.10–1.20), and the presence of hypertension (OR 2.10, 95% CI 1.59–2.76) and diabetes (OR 2.17, 95% CI 1.53–3.07). In the age-adjusted logistic regression analysis, the presence of diabetes (OR 1.54, 95% CI 1.07–2.22) and the presence of myopia (OR 1.69, 95% CI 1.24–2.31) were significantly associated with OAG (Table 2).

In participants who had natural menopause, old age (OR 1.04 per year, 95% CI 1.02–1.05), early menopause (OR 2.40, 95% CI 1.50–3.83), parity (OR 1.08 per pregnancy, 95% CI 1.03–1.14), the presence of hypertension (OR 1.57, 95% CI 1.15–2.13), and myopia (OR 1.53, 95% CI 1.06–2.21) were significantly associated with OAG in univariate logistic regression analyses. Age-adjusted logistic regression analysis found that early menopause (OR 1.88, 95% CI 1.11–3.19) and myopia (OR 1.59, 95% CI 1.10–2.32) were significantly associated with OAG.

In multivariate logistic regression analysis after adjusting for all potential confounding factors, early menopause was significantly associated with OAG only in participants with natural menopause (OR 1.60, 95% CI 0.94–2.73 in all participants versus OR 2.28, 95% CI 1.17–4.46 in participants with natural menopause) (Table 3). Multivariate logistic regression analysis did not show a statistically significant association between early menarche and OAG (OR 1.01, 95% CI 0.38–2.65 in all participants and OR 1.10, 95% CI 0.39–3.15 in participants with natural menopause) or between parity and OAG (OR 1.02, 95% CI 0.95–1.10 in all participants and OR 1.02, 95% CI 0.95–1.08 in participants with natural menopause).

## 4. Discussion

According to a previous population-based prevalence study in Korea using KNHANES data, the primary OAG prevalences for the different age groups, 40 to 49 years, 50 to 59 years, 60 to 69 years, 70 to 79 years, and 80 years or older, were 3.6%, 5.9%, 7.3%, 8.8%, and 10.5% in men, and 2.0%, 3.3%, 5.1%, 7.7%, and 8.1% in women, respectively [16]. Our data showed similar prevalences in each age group of female. Although our study did not reveal the incidence of OAG, in a previous cohort study in Korea, the 5-year incidences of primary OAG among women aged 40 to 49, 50 to 59, 60 to 69, and 70 years or older were 0.32%, 0.50%, 1.45%, and 3.45%, respectively [17]. Several risk factors of OAG have been mentioned in these epidemiological studies in Korea. However, population-based studies of female reproductive factors as risk factors for OAG were lacking. In this population-based study of Korean women, we found that early natural menopause was associated with an increased risk of OAG. There was no significant association between OAG and age at menarche, reproductive duration, parity, or use of OC or HRT among Korean women.

Our findings agree with many previous population-based studies showing that early menopause might increase the risk of OAG. In the Rotterdam Study, the first population-based study about OAG and early menopause, the risk of OAG was higher in women who entered menopause before 45 years of age (odds ratio (OR) 2.6; 95% CI 1.5–4.8) [7]. According to secondary analysis of the Nurses' Health Study (NHS), the risk of OAG was 50% lower in women who underwent menopause at age ≥ 54 years than at age < 54 years (relative risk (RR) 0.53; 95% CI 0.32–0.89) over more than 20 years of follow-up [4]. In the Singapore Malay Eye study, women who had menopause at a younger age were more likely to have glaucoma (before age 53 years, OR 3.54; 95% CI 1.24–10.12) [9]. On the contrary, the risk of OAG was not associated with early menopause in the Blue Mountains Study [5], and the Aravind Comprehensive Eye Survey also did not find significant associations between early menopause and the risk of OAG [8].

In our study, both early and late menarche were not associated with the risk of OAG. Some previous population-based studies have reported that early menarche may decrease the risk of OAG because of the longer duration of estrogen exposure over a lifetime. In the Blue Mountains Study, the risk of OAG was significantly higher in women who reported late menarche (after age 13 years, OR 2.0; 95% CI 1.0–3.9) [5]. Recently, a population-based study in the United States also found that older age at menarche was significantly associated with a higher prevalence of self-reported glaucoma or ocular hypertension using data from the National Health and Nutrition Examination Survey (NHANES) (OR 1.13; 95% CI 1.03–1.22) [13]. However, in a cohort study with more than 25 years of follow-up in the NHS, there was no significant relationship between age at menarche and OAG [18], in agreement with our findings.

We assumed that estrogen in the menopausal period may help protect against OAG. Although the exact mechanism of the protective effect of estrogen has not been elucidated, some mechanisms have been suggested. Estrogen enhances ocular blood flow, and it has been demonstrated that aging and age-related declines in female sex hormones negatively affect ocular blood flow [18]. Toker et al. showed that serum estrogen has beneficial effects on blood flow velocities and resistive indices in the retrobulbar arteries [6], which might result from vascular smooth muscle relaxation [19–21]. Estrogen is also known to reduce IOP in women [22–26]. 17*β*-estradiol augments the activity of endothelial nitric oxide synthase (NOS) [27], which in turn mediates vasodilation and vascular tone to modulate blood flow to the optic nerve [28] and might potentially influence IOP by regulating both aqueous production and aqueous outflow through receptors located in the ciliary body and the outflow system [25, 29]. Additionally, the neuroprotective effects of estrogen on the optic nerve are supported by clinical, epidemiological, and basic science evidence [30–37].

Among female reproductive factors, only early menopause was significantly associated with OAG in this study, while age at menarche was not. We offer several reasons for these findings in our study. First, the protective role of estrogen against OAG is more effective in menopausal women because the prevalence of OAG is much higher in old age, and the development of glaucoma is a degenerative aging process. A recent clinical study suggested that in postmenopausal women, estrogen deficiency is a causative factor for increased susceptibility to glaucomatous damage with age [30] and use of HRT protects the retinal nerve fiber layer [38]. Previous studies have also shown that postmenopausal women with glaucoma suffer from more serious damage of optic nerves than younger women with glaucoma, even at the same level of IOP [39]. Second, the relationship between myopia and age at menarche may influence our results. According to a recent Korean population-based study, later age at menarche is associated with a decreased risk of moderate to high myopia [40], and the effects of female sex hormones on ocular structures (e.g., the change of axial length) may mediate this relationship. Late menarche reduces the duration of estrogen exposure, which may lead to the development of OAG, as reported in Western studies, while decreased risk of myopia development in late menarche may help protect against OAG. Therefore, in Korean women, age at menarche was not associated with OAG due to these conflicting reasons.

The use of OC or HRT was not associated with the risk of OAG in this study. In recent Caucasian population-based studies, OC use for more than 3 years was associated with a greater risk of self-reported glaucoma or ocular hypertension [13], and OC use for more than 5 years was associated with a modestly increased risk of OAG [18]. In addition, HRT improves ocular blood flow in postmenopausal women [38], reduces vascular resistance in the central retinal artery and posterior ciliary artery [25, 41], and decreases IOP [42, 43]. In some studies, estrogen-only treatment was more effective at lowering IOP than the combination of estrogen and progesterone [24]. In contrast, in the Rotterdam [7] and Blue Mountains Eye studies [5], the risk of OAG did not significantly reduce the odds in Caucasian women who had used HRT. Similarly, in the Singapore Malay Eye study and a recent Korean study regarding ocular benefits after HRT, no significant association was observed between the use of HRT and OAG [9, 44].

The strength of our study is that the data were collected from a nationwide survey in Korea. Unlike previous similar Asian studies [8, 9], ours is the first nationwide general population-based study of the association between female reproductive factors and OAG conducted in Asian women. Second, the KNHANES data underwent rigorous quality control of study procedures. Third, OAG was diagnosed in this study according to standard protocols, allowing us to include all undiagnosed OAG patients; in some studies, the OAG group has only included those previously diagnosed with OAG.

Several issues should be considered during interpretation of our results. First, because the KNHANES used self-reported interview data for age at menopause and menarche, as well as the cause of menopause, recall bias could be important. Second, the retrospective and cross-sectional design makes it difficult to explain the causality between female reproductive factors and risk of OAG. Future studies are needed to assess cause-effect relationships in the Korean population. Third, although the KNHANES provides a large, robust database, relatively few participants fit our criteria, limiting the power of the analysis. A larger study could better elucidate the role of female reproductive factors. Fourth, the definition of open angle was made basically by Van Herick method rather than standard gonioscopy, which might misclassify some angle closure glaucoma (such as cases with plateau iris) as OAG. Finally, the KNHANES did not distinguish between estrogen-only treatment or a combination of estrogen and progestin for the type of HRT.

Previous Western studies have found that early menopause and late menarche, as well as the use of OC or HRT in some studies, are associated with OAG. Our data from a representative population of Korean women indicate that, among female reproductive factors, only early menopause was associated with OAG, consistent with the results of a previous study in Singapore. This result may suggest not only a protective effect of estrogen against the development of OAG in old age, but also genetic or ethnic differences between Western and Asian women. Causative relationships and the role of other female reproductive factors in OAG in Asians should be confirmed by additional studies.

## Figures and Tables

**Table 1:** Descriptive statistics for demographics and clinical characteristics of the study participants.

	Open-angle glaucoma (*n*=397)	Non-open-angle glaucoma (*n*=6463)	*P* value
Age, years	62.7 ± 0.8	55.8 ± 0.2	<0.001^a^
Age at menarche, years			
≤12 years (%)	5.5 ± 1.8	5.8 ± 0.4	0.224^a^
13–16 years (%)	58.4 ± 3.1	63.7 ± 0.9	
≥17 years (%)	36.1 ± 2.9	30.5 ± 0.8	
Age at menopause, years	49.0 ± 0.3	48.7 ± 0.1	0.700^b^
Early menopause (<45 years) (%)	19.1 ± 3.1	13.2 ± 0.7	0.033^a^
Natural menopause (%)	90.2 ± 2.0	85.8 ± 0.7	0.07^a^
Parity	5.0 ± 0.1	4.3 ± 0.0	0.013^b^
Reproductive years, years	32.8 ± 0.4	32.7 ± 0.1	0.898^b^
Hypertension (%)	53.8 ± 3.4	35.7 ± 0.8	<0.001^a^
Diabetes mellitus (%)	20.4 ± 2.8	10.5 ± 0.5	<0.001^a^
Oral contraceptives use (ever, %)	16.0 ± 2.3	17.7 ± 0.6	0.515^a^
≥3 years (%)	23.0 ± 7.0	21.9 ± 1.6	0.882^a^
Hormone replacement (ever, %)	9.9 ± 1.9	10.9 ± 0.5	0.625^a^
Myopia (%)	26.6 ± 2.9	23.0 ± 0.7	0.209^a^
Body mass index	23.9 ± 0.2	24.0 ± 0.1	0.545^b^
Lactation (ever, %)	85.1 ± 2.6	82.5 ± 0.6	0.355^a^

Values are presented as weighted mean ± standard error. *P* value is calculated by the chi-square test^a^ or independent *t*-test^b^ within the complex sample analysis method.

**Table 2:** Univariate and age-adjusted logistic regression analyses for the association between factors and open-angle glaucoma in all participants and participants with natural menopause.

All participants	Univariate		Age-adjusted	
	OR (95% CI)	*P* value	OR (95% CI)	*P* value
Age, per year	1.04 (1.03–1.06)	<0.001		
Age at menarche, years				
13–16 years	Reference	0.145	Reference	0.126
≤12 years	1.03 (0.53–2.03)		1.39 (0.70–2.74)	
≥17 years	1.29 (1.00–1.67)		0.80 (0.62–1.04)	
Early menopause (<45 years) (%)	1.56 (1.03–2.35)	0.035	1.41 (0.91–2.16)	0.122
Parity	1.15 (1.10–1.20)	<0.001	1.01 (0.96–1.07)	0.627
Reproductive years	1.00 (0.97–1.03)	0.804	1.01 (0.98–1.04)	0.396
Hypertension (%)	2.10 (1.59–2.76)	<0.001	1.33 (0.99–1.80)	0.015
Diabetes mellitus (%)	2.17 (1.53–3.07)	<0.001	1.54 (1.07–2.22)	0.021
Oral contraceptives use (ever, %)	0.89 (0.63–1.26)	0.515	0.75 (0.52–1.06)	0.099
≥3 years	1.06 (0.48–2.36)	0.882	0.94 (0.42–2.11)	0.875
Hormone replacement (ever, %)	0.90 (0.58–1.39)	0.625	0.90 (0.58–1.39)	0.623
Myopia (%)	1.21 (0.90–1.64)	0.210	1.69 (1.24–2.31)	0.001
Body mass index	0.99 (0.95–1.03)	0.552	0.98 (0.95–1.02)	0.390
Lactation (ever, %)	1.21 (0.85–1.83)	0.259	0.68 (0.45–1.04)	0.106

*Participants with natural menopause*				
Age, per year	1.04 (1.02–1.05)	<0.001		
Age at menarche, years				
13–16 years	Reference	0.335	Reference	0.041
≤12 years	1.70 (0.74–3.90)		1.94 (0.85–4.47)	
≥17 years	0.94 (0.70–1.26)		0.79 (0.59–1.06)	
Early menopause (<45 years) (%)	2.40 (1.50–3.83)	<0.001	1.88 (1.11–3.19)	0.019
Number of parity	1.08 (1.03–1.14)	0.003	1.01 (0.95–1.07)	0.808
Reproductive years, years	1.00 (0.96–1.03)	0.846	1.01 (0.98–1.05)	0.439
Hypertension (%)	1.57 (1.15–2.13)	0.005	1.24 (0.89–1.73)	0.200
Diabetes mellitus (%)	1.47 (0.95–2.28)	0.085	1.27 (0.82–1.98)	0.114
Oral contraceptives use (ever, %)	0.82 (0.56–1.19)	0.286	0.80 (0.55–1.16)	0.239
≥3 years	1.04 (0.45–2.40)	0.936	1.02 (0.44–2.37)	0.961
Hormone replacement (ever, %)	0.86 (0.54–1.37)	0.522	1.06 (0.66–1.70)	0.805
Myopia (%)	1.53 (1.06–2.21)	0.022	1.59 (1.10–2.32)	0.015
Body mass index	0.98 (0.94–1.03)	0.486	0.99 (0.94–1.03)	0.587
Lactation (ever, %)	0.92 (0.53–1.60)	0.775	0.68 (0.38–1.19)	0.176

CI = confidence interval.

**Table 3:** Multivariate logistic regression analyses for the association between open-angle glaucoma and age at menarche, early menopause, and parity.

		All		Natural menopause	
		OR (95% CI)	*P* value	OR (95% CI)	*P* value
Age at menarche, years					
13–16 years	Model I	Reference	0.506	Reference	0.397
≤12 years		0.99 (0.45–2.15)		1.10 (0.40–3.02)	
≥17 years		0.83 (0.60–1.14)		0.79 (0.56–1.13)	
13–16 years	Model II	Reference	0.425	Reference	0.279
≤12 years		1.01 (0.38–2.65)		1.10 (0.39–3.15)	
≥17 years		0.80 (0.56–1.13)		0.75 (0.52–1.09)	

Early menopause <45 years					
	Model I	1.50 (0.96–2.34)	0.073	2.05 (1.22–3.50)	0.009
	Model II	1.60 (0.94–2.73)	0.082	2.28 (1.17–4.46)	0.016

Parity					
	Model I	1.03 (0.97–1.09)	0.415	1.02 (0.96–1.08)	0.556
	Model II	1.02 (0.95–1.10)	0.631	1.02 (0.94–1.11)	0.686

CI = confidence interval. Model I, adjusted for age, diabetes mellitus, hypertension, myopia, body mass index, oral contraceptive use, and hormone replacement. Model II, adjusted for age, diabetes mellitus, hypertension, myopia, body mass index, oral contraceptive use, hormone replacement, and female reproductive factors (age at menarche, early menopause, lactation history, and parity).

## Data Availability

The data used to support the findings of this study are available from the corresponding author upon request.
